# Physical Activity, Sedentary Time, and Psychosocial Functioning among Adults with Cancer: A Scoping Review

**DOI:** 10.3390/ijerph21020225

**Published:** 2024-02-14

**Authors:** Arianne Côté, Paule Miquelon, Claudia Trudel-Fitzgerald

**Affiliations:** 1 Department of Psychology, Université du Québec à Trois-Rivières, Trois-Rivières, QC G8Z 4M3, Canada; paule.miquelon@uqtr.ca (P.M.); claudia.trudel-fitzgerald@uqtr.ca (C.T.-F.); 2 Research Center of Institut Universitaire en Santé Mentale de Montréal, Montréal, QC H1N 3V2, Canada; 3 Lee Kum Sheung Center for Health and Happiness, Harvard T.H. Chan School of Public Health, Boston, MA 02115, USA

**Keywords:** physical activity, sedentary behavior, post-treatment, psychological functioning, quality of life, mental health, oncology, cancer

## Abstract

The post-treatment period (after the completion of primary cancer treatment) is a phase during which adults with cancer are particularly vulnerable to the physical and psychological side effects of treatment. Adopting healthy lifestyle habits during this time is essential to mitigate these effects. This scoping review investigated the associations of physical activity (PA) and sedentary time (ST) with two post-treatment psychosocial indicators among adults with cancer: psychological functioning and quality of life (QoL). An exhaustive search was performed in January 2023 across five databases, namely APA PsycInfo, MedLine, SPORTDiscuss, SCOPUS, and CINAHL, adhering to PRISMA guidelines for scoping reviews. Twenty articles met the inclusion criteria; 16 used a cross-sectional design, while 4 used a longitudinal one. PA and ST were assessed mainly with accelerometers (*n* = 17), and psychosocial indicators with self-reported questionnaires (*n* = 20). Most studies linked higher PA levels to reduced anxiety (*n* = 3) and depression (*n* = 4) symptoms, and elevated ST to higher psychological symptoms (*n* = 3). Opposite associations were observed for QoL (*n* = 5). Altogether, PA appeared to be more strongly related to psychological functioning and QoL than ST. This scoping review highlights associations of PA and ST with psychological functioning and QoL among adults with cancer in the post-treatment period. However, future studies must prioritize longitudinal designs to establish directionality.

## 1. Background

Cancer is the leading cause of death in Canada, ahead of cardiovascular disease [1]. Fortunately, thanks to advances in early detection and treatment, the five-year survival rate for most cancers has risen from 34% in the mid-1970s to 67% in 2016 [2]. Although treatments’ effectiveness has improved, cancer survivors are often left with persistent side effects from chemotherapy, radiotherapy, and surgery, including depression, anxiety, and fatigue symptoms, as well as decreased physical function, which can reduce quality of life (QoL) [3]. For instance, a national Canadian survey revealed that 68% of cancer survivors between one and three years post-treatment reported suffering from anxiety, stress, or worries about cancer recurrence, while 46% experienced depression, sadness, or loss of interest in everyday activities [4].

Results of several systematic reviews and meta-analyses support the important role played by physical activity (PA) in improving the psychological functioning and QoL of cancer survivors (e.g., [5,6,7]). In this respect, the American Cancer Society recommends the same guidelines for patients with cancer who have completed their treatment [8] as the ones advised by the World Health Organization for adults of the general population [9], which is to engage in ≥150 min of moderate-intensity PA or ≥75 min of vigorous-intensity PA per week.

Conversely, sedentary time (ST; i.e., any waking behaviour characterized by an energy expenditure ≤1.5 METs while in a sitting or reclining posture [10]) is an independent risk factor for cancer-related mortality and morbidity [10]. The distinction between ST and physical inactivity (i.e., not adhering to PA guidelines) is important, as it implies that PA recommendations can be met while simultaneously leading a sedentary lifestyle, or vice versa [11]. Existing evidence on ST specifically suggests that excessive ST is associated with greater anxiety, depression, fatigue, and pain symptoms, as well as poorer mental well-being, and reduced QoL in adults with cancer [12,13,14,15,16,17].

Despite the benefits of increased PA and reduced ST on such psychosocial indicators, a recent review found that cancer survivors spent, on average, only 3% of their time practicing moderate-to-vigorous PA (MVPA), while they spent 66% of their time being sedentary [18]. Existing longitudinal [19] and cross-sectional [20] studies also show that even if a person achieves PA recommendations, PA’s positive association with psychological functioning and QoL should diminish if too much ST is spent during the day. For these reasons, it is particularly relevant to examine PA and ST simultaneously rather than separately in relation to psychosocial indicators in oncology.

The post-treatment period (i.e., after the completion of primary cancer treatment [21]) is a phase during which adults with cancer are particularly vulnerable to the physical and psychological side effects of treatment [22,23,24]. During this phase, the adoption of healthy lifestyle habits is crucial to countering these effects. However, to date, the concurrent associations of PA and ST with psychological functioning and QoL during this period remain poorly understood in adults with cancer [25]. Indeed, while a few reviews have investigated the associations between PA and psychosocial functioning in this population (e.g., [26,27]), ST is a much more recent concept that is often misinterpreted as physical inactivity rather than a distinct behaviour with unique health implications. To date, most reviews studying ST in oncology examined its associations with cancer risk (e.g., [28,29]) or mortality (e.g., [11,30]). Therefore, no study has yet examined the concurrent associations of PA and ST with psychological functioning and QoL among cancer survivors during the post-treatment period. It thus appears essential to conduct a scoping review to systematically map the available evidence, as well as to identify any research gaps and inform the direction of future studies. Therefore, the aim of this paper was to address the following research question: What are the unique and concurrent associations of PA and ST with the post-treatment psychological functioning and QoL of adults with cancer?

## 2. Methods

### 2.1. Inclusion and Exclusion Criteria

Studies were eligible for inclusion if they were (1) published in English, (2) published in peer-reviewed journals, (3) published in the year 2000 or after, and (4) conducted among participants aged 18 and over, and if they had (5) focused on participants who had been diagnosed with cancer in adulthood and completed active oncological treatment for it, (6) included a measure of PA, (7) included a measure of ST, (8) included at least one measure associated with psychological functioning and/or QoL, and (9) analyzed both the associations of PA and ST with psychological functioning and/or QoL.

Regarding criterion number 5, studies conducted with patients currently on hormone treatment were included, as this type of treatment does not have the same impact on patients’ daily lives and functioning as chemotherapy, radiotherapy, and surgery [31]. More specifically, hormone therapy consists of daily medication and therefore does not involve frequent hospital visits, which may impede an individual’s ability to engage in PA, as is the case with chemotherapy, radiotherapy, and surgery. This criterion has been used in previous studies (e.g., [32]). With respect to criterion number 3, the year 2000 was chosen as a cut point to include as many relevant studies as possible while avoiding a very large number of articles failing to meet inclusion criterion number 7. Indeed, a terminology consensus to highlight the differences between PA and ST was only established in 2017 by the Sedentary Behaviour Research Network [10]. Prior to that, and as mentioned above, sedentary behaviour was often considered as physical inactivity rather than a distinct behaviour with unique health implications. However, some studies published in the early 2000s have already distinguished between the concepts of PA and ST (e.g., [33,34,35]), which is why we chose to extend our research to 2000.

Alternatively, studies were excluded if participants had a current severe mental or physical health diagnosis other than cancer. This exclusion criterion was implemented given that these conditions can alter psychological functioning [36] or hinder the practice of PA [37], without being attributable to the cancer itself. Moreover, grey literature (e.g., theses and dissertations, reports, and conference papers), case studies, exclusive qualitative research, meta-analyses, and systematic reviews were also excluded, as advised elsewhere [38,39].

### 2.2. Data Sources and Search Strategy

Identification and selection of the studies were conducted according to guidelines from the PRISMA extension for scoping reviews [40]. We searched five databases, namely APA PsycInfo, MedLine, SPORTDiscuss, SCOPUS, and CINAHL, in January 2023, using the search strategy shown in Table 1. A specialized librarian was consulted to ensure that all relevant keywords were identified.

### 2.3. Study Selection Process

All studies were downloaded as Endnote files from their respective databases, and duplicate records were removed. Study selection was made in two stages by a researcher (AC): first, titles and abstracts were screened, and then full texts of the articles were reviewed for inclusion. At the end of the selection process, two other researchers (PM and CTF) reviewed the selected articles, and a consensus was reached between all three researchers.

**Figure 1:**
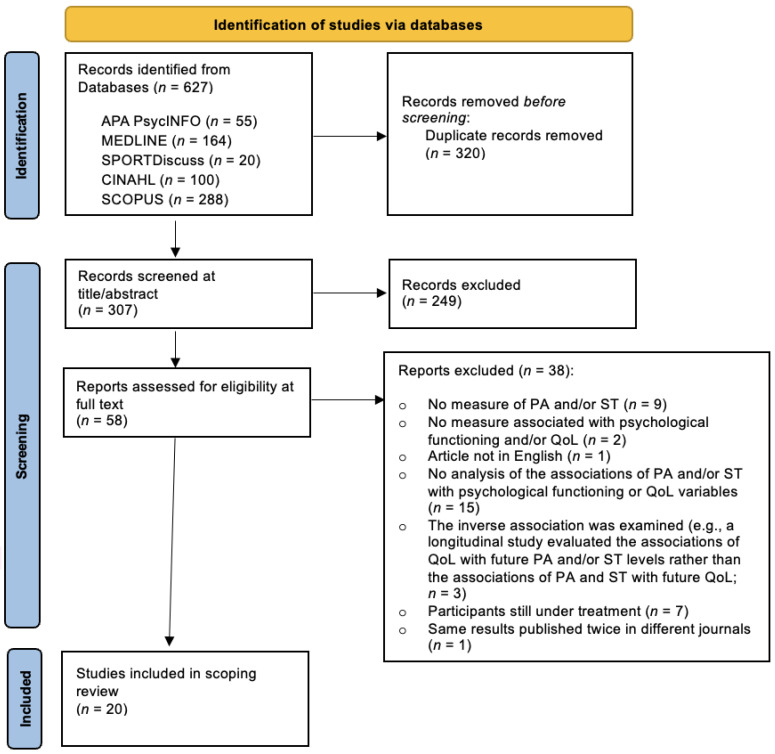
Process of identification and inclusion of studies—PRISMA diagram flow.

The initial search yielded 627 articles. After removing duplicates, the titles and abstracts of 307 articles were reviewed, resulting in 58 primary research articles for full-text assessment. At this stage, 38 articles were excluded for the following reasons: (1) they included no measure of PA and/or ST (*n* = 9), (2) they included no measure of psychological functioning and/or QoL (*n* = 2), (3) they were not written in English (*n* = 1), (4) there was no analysis of the associations of PA and ST with psychological functioning or QoL (*n* = 15), (5) the inverse association was examined (e.g., a longitudinal study evaluated the associations of QoL with future PA and/or ST levels rather than the associations of PA and ST with future QoL; *n* = 3), (6) they included participants who were still under treatment (*n* = 7), and (7) the same results were published twice in different journals (*n* = 1). The review therefore included 20 articles (see Figure 1).

### 2.4. Data Extraction

The results were extracted by a single researcher (AC) into an Excel data chart, which was developed by the three authors (AC, PM, and CTF). The following data were extracted: article’s first author and year of publication, study design, PA and ST measures, psychological functioning and/or QoL measures, sample size, country of study, sex, mean age, type(s) of cancer, type(s) of treatment received, and time since the end of treatment (see Table 2), as well as specific findings for each included study (see Supplementary Table S1). Data extraction was verified by two researchers (PM and CTF) to reach 100% agreement. Quality appraisal and/or risk of bias assessments were not conducted, as they are deemed beyond the aim of scoping reviews [41].

## 3. Results

### 3.1. Descriptive Characteristics of Studies

For the 20 studies included in this scoping review (Table 2), the publication years ranged from 2013 to 2022. The included studies spanned many different countries: seven from Canada, five from the United States, five from Australia, two from the Netherlands, and one each from China, Malaysia, and Italy. Of these, 16 studies used a cross-sectional design, while four used a longitudinal design; all (*n* = 20) were observational.

### 3.2. Targeted Population

The sample sizes ranged from 20 to 8466 patients with cancer, for a total of 13,129 participants (7280 women), with a mean age ranging from 50–78 years. Most studies targeted a specific cancer site, namely breast (*n* = 8), colon (*n* = 2), lung (*n* = 2), colorectal (*n* = 2), prostate (*n* = 1), ovarian (*n* = 1), and non-Hodgkin lymphoma (*n* = 1), while three studies combined many cancer sites.

Not all included studies reported the mean time since completion of oncological treatments, but all required participants to have completed their primary treatments as part of their inclusion criteria (however, studies with patients who were still on hormone therapy were retained, as described in Section 2.1 above) or to have had a mean time since diagnosis of over 18 months. Indeed, we considered studies with a mean time since diagnosis of over 18 months to be carried out within the post-treatment period and therefore included them, given that chemotherapy and radiotherapy last, on average, 3 to 6 months and 3 to 9 weeks, respectively [31]. More specifically, five studies reported mean time since completion of treatment, which ranged from 3–31 months, whereas 11 studies reported only mean time since diagnosis, which ranged from 19 months to seven years. Out of the remaining four studies, three used a timeframe ranging from six weeks to 48 months post-treatment, whereas the last one did not provide a timeframe but required participants to have completed their oncological treatment.

### 3.3. Assessment of PA and ST

Most included studies assessed PA and ST using an objective measure (*n =* 17), the most popular being the ActiGraph accelerometer (*n =* 12), a device allowing participants’ PA and ST to be captured via mechanical measurements of core and limb movements [42]. Indeed, the ActiGraph is the type of accelerometer most frequently used to objectively assess PA and ST in cancer research [43]. Five other studies measured PA and ST using another type of accelerometer, which was either the SenseWear Pro3 or SenseWear Mini armbands (*n =* 1), the activPAL (*n =* 2), or the triaxial MOX activity monitor (*n =* 2). The accelerometer was mainly worn on the hip (*n =* 11) and always for seven consecutive days. The remaining studies that leveraged a subjective PA/ST measure used either the International Physical Activity Questionnaire (IPAQ; *n =* 1), the Leisure Time Exercise Questionnaire (*n* = 1), the Short QUestionnaire to ASsess Health-enhancing physical activity (known as SQUASH; *n =* 1) or homemade questions formulated specifically for the study (*n =* 1).

**Table 2:** Summary table of included studies in the scoping review.

First Author, Year of Publication	Study Design	PA and ST Measures	Psychological Functioning and/or QoL Measures	Participants				
				Sample Size and Country	Sex	Mean Age (±SD)	Cancer and Treatment Types	Mean Time since End of Treatment (±SD)
Di Blasio et al., 2022 [44]	Cross-sectional	SenseWear Pro3 or SenseWear Mini armband accelerometer (worn on a wrist for 7 days)	Anxiety and depression: HADS	219 (Italy)	Women	50.98 (±6.28) Range: 30–60	Breast Surgery (all) Hormone therapy (all)	Not reported but 6 to 48 months after breast surgery and current hormone therapy ^a^
Doré et al., 2022 [17]	Longitudinal over 48 months (Every 3 months during the first year, and then after 24 and 48 months)	ActiGraph GT3X accelerometer (worn on a hip for 7 days every 3 months during the first year, and then after 24 and 48 months)	Depression: CES-D	199 (Canada)	Women	55.0 (±11.0) Range: 28–79	Breast (Stages I–III) Lymph or axillary node dissection (*n* = 116) Lumpectomy (*n* = 119) Single mastectomy (*n* = 56) Double mastectomy (*n* = 34) Chemotherapy (*n* = 128) Radiotherapy (*n* = 176) Hormonal therapy (*n* = 101)	3.5 months (±2.3)
D’Silva et al., 2018 [12]	Cross-sectional	ActiGraph GT3X accelerometer (worn on a hip for 7 days)	QoL: FACT-Lung	127 (Canada)	73 women and 54 men	71.4 (±9)	Lung (Stages I–III) Surgery (*n* = 82) Surgery and adjuvant chemotherapy (*n* = 32) Radical (*n* = 16) Palliative (*n* = 10 ^b^) None (*n* = 3 ^c^)	Not reported but mean time since diagnosis = 76.4 months (±47.0)
Floor Kenkhuis et al., 2021 [45]	Longitudinal over 24 months (6 weeks, 6 months, 12 months, and 24 months post-treatment)	PA: SQUASH ST: triaxial MOX accelerometer (worn on a thigh for 7 days at 6 weeks, and 6, 12, and 24 months post-treatment)	QoL: EORTC QLQ-C30	397 (The Netherlands)	270 men and 126 women	67.0 (±9.1)	Colorectal (Stages I–III) Surgery (*n* = 354) Chemotherapy (*n* = 155) Radiotherapy (*n* = 101) Stoma (*n* = 110)	Not reported but 6 weeks to 24 months
Gaskin et al., 2016 [32]	Cross-sectional	ActiGraph GT1M accelerometer (worn on a hip for 7 days)	QoL: EORTC QLQ-C30 + EORTC QLQ- PR25 Anxiety: MAX-PC Depression: CES-D	98 (Australia)	Men	67.3 (±8.0)	Prostate (Stages I–III) Surgery only (*n* = 37) Radiotherapy only (*n* = 14) Surgery and radiotherapy (*n* = 27) Hormonotherapy with surgery and/or radiotherapy (*n* = 20)	26.1 weeks (±10.1)
Hartman et al., 2017 [46]	Cross-sectional	ActiGraph GT3X+ accelerometer (worn on a hip for 7 days)	QoL: SF-36	134 (USA)	Women	62.6 (±6.6)	Breast (Stages I–III) Surgery (all) Chemotherapy (*n* = 65) Endocrine therapy (*n* = 93)	Not reported but mean time since diagnosis = 2.1 years (±1.2)
Hidde et al., 2022 [47]	Cross-sectional	activPAL accelerometer (worn on a wrist for 7 days)	QoL: FACT-G	73 (USA)	55 women and 18 men	53 (±13.0)	Breast (*n* = 22) Colorectal (*n* = 24) Leukemia/Lymphoma (*n* = 7) Other (*n* = 20) (Stages 0–IV) Surgery (*n* = 66) Chemotherapy (*n* = 54) Radiation (*n* = 38) Other (*n* = 12)	Between 16.3 months and 31.2 months depending on the treatment
Nurnazahiah et al., 2022 [48]	Cross-sectional	ActivPAL3TM accelerometer (worn on a thigh for 7 days)	QoL: EORTC QLQ–C30	83 (Malaysia)	Women	52.8 (±7.8)	Breast (Stages I–III) Surgery (*n* = 83) Chemotherapy (*n* = 83) Radiotherapy (*n* = 73)	Not reported but mean time since diagnosis = 6.84 years (±4.13)
Phillips et al., 2015 [49]	Longitudinal over 6 months (Baseline and 6 months later)	ActiGraph accelerometer (worn on a hip for 7 days at baseline only)	QoL: FACT-B Anxiety and depression: HADS	358 (USA)	Women	56.4 (±9.0)	Breast (Stages 0–IV) Surgery + radiotherapy + chemotherapy (*n* = 142) Surgery + radiotherapy (*n* = 101) Surgery + chemotherapy (*n* = 60) Surgery only (*n* = 55)	Not reported but mean time since diagnosis = 81.7 months (±67.7)
Rees-Punia et al., 2020 [25]	Cross-sectional	Questions created specifically for the aim of the study	Global mental health: PROMIS	7966 (USA)	3395 women and 4571 men	78.3 (±5.4)	Colorectal (*n* = 810) Lung (*n* = 282) Breast (*n* = 1708) Endometrial (*n* = 308) Prostate (*n* = 3089) Other (*n* = 1769) * Treatment types were neither presented nor detailed in the study.	Not reported but mean time since diagnosis = 2.9 years (±1.2) in the first group (1–5 years after diagnosis) and 7.5 years (±1.4) in the second group (6–10 years after diagnosis)
Roekel et al., 2016 [50]	Cross-sectional	triaxial MOX accelerometer (worn on a thigh for 7 days)	QoL: EORTC QLQ-C30 Anxiety and depression: HADS	145 (The Netherlands)	54 women and 91 men	70.0 (±8.7)	Colorectal (Stages I–III) Surgery (*n* = 139) Chemotherapy (*n* = 75) Radiotherapy (*n* = 55)	Not reported but mean time since diagnosis = 5.7 years (±1.9)
Schofield et al., 2018 [51]	Cross-sectional	ActiGraph GT3X+ accelerometer (worn on a hip for 7 days)	QoL: SF-36	20 (Australia)	Women	63.2 (±8.9)	Ovarian (Stages III–IV) Surgery (all) Chemotherapy (all)	5.3 months
Trinh et al., 2015 [52]	Cross-sectional	ActiGraph GT3X accelerometer (worn for 7 days; no information regarding where the accelerometer was worn available in the article)	Depression: CES-10	195 (Canada)	Women	55.0 (±11.0)	Breast (Stages I–III) Lumpectomy (*n* = 117) Single mastectomy (*n* = 54) Double mastectomy (*n* = 31) Chemotherapy (*n* = 125) Radiotherapy (n = 174) Hormonal therapy (*n* = 98)	3.5 months (±2.4)
Vallance et al., 2014 [53]	Cross-sectional	ActiGraph GT3X+ accelerometer (worn on a hip for 7 days)	QoL: FACT-C	178 (Canada and Australia)	79 women and 99 men	64.3 (±10.3)	Colon (Stages I–III) Chemotherapy (*n* = 80) Others ^d^	Not reported but mean time since diagnosis = 18.9 months (±4.4)
Vallance et al., 2015 [54]	Cross-sectional	ActiGraph GT3X+ accelerometer (worn on a hip for 7 days)	Depression: PHQ-9 State anxiety: SAI Satisfaction with life: SWLS	180 (Canada and Australia)	81 women and 99 men	64.3 (±10.3)	Colon (Stages I–IV) Chemotherapy (*n* = 81) Others ^d^	Not reported but mean time since diagnosis = 18.8 months (±4.4)
Vallance et al., 2017 [55]	Cross-sectional	ActiGraph GT3X accelerometer (worn on a hip for 7 days)	QoL: FACT-G	156 (Australia)	76 women and 80 men	62.2 (±12.9) Range: 22–82	Non-Hodgkin lymphoma Chemotherapy only (*n* = 48) Other treatment only (*n* = 15) Chemotherapy and 1 other treatment (*n* = 59) Chemotherapy and 2+ other treatments (*n* = 18) None (*n* = 15 ^e^)	Not reported but mean time since diagnosis = 35.1 months (±11.6)
Vallance et al., 2018 [56]	Cross-sectional	ActiGraph GT3X accelerometer (worn on a hip for 7 days)	Depression: PHQ-9 State anxiety: SAI Psychological growth: PTGI Satisfaction with life: SWLS	127 (Canada)	73 women and 54 men	71.49 (±9.0)	Lung (Stages I–IV) Surgery (*n* = 82) Radical (*n* = 32) Palliative (*n* = 10) None (*n* = 3 ^f^)	Not reported but mean time since diagnosis = 76.4 months (±47.0)
Welch et al., 2019 [57]	Cross-sectional	ActiGraph GT1M and GT3X accelerometer (worn on a hip for 7 days)	QoL: FACT-B Anxiety and depression: HADS	753 (USA)	Women	56.4 (±9.5)	Breast Chemotherapy (*n* = 462) Radiation (*n* = 513) Chemotherapy + radiation (*n* = 306)	Not reported but mean time since diagnosis = 7.0 years (±5.9)
Wrosch et al., 2013 [58]	Longitudinal over 3 months (Baseline and 3 months later)	Adapted version of the Leisure Time Exercise Questionnaire	Emotional well-being: POMS	176 (Canada)	Women	54.86 (±10.83)	Breast Lumpectomy (*n* = 131) Lymph node dissection (*n* = 118) Single mastectomy (*n* = 60) Double mastectomy (*n* = 45) Chemotherapy (*n* = 136) Radiotherapy (*n* = 153) Hormone therapy (*n* = 108)	2.89 months (±2.86)
Yan et al., 2021 [59]	Cross-sectional	IPAQ	QoL: EORTC QLQ-C30	1546 (China)	1131 women and 415 men	Not reported but 53.56% were 60 years old and above	Not reported (all ^g^)	Not reported, but participants had to have completed initial treatment ^h^

^a^ As mentioned in Section 2.1 above, studies conducted with patients currently on hormone treatment were included, even though studies with patients receiving other types of treatment were excluded, as this specific type of treatment does not have the same impact on patients’ daily lives and functioning as chemotherapy, radiotherapy, and surgery [31]. ^b^ We considered excluding this study, given that some participants were in palliative care and therefore probably unable to practice PA. However, we decided to include it since only 8% of the sample was in palliative care. ^c^ We chose to include this study, even though some participants did not meet inclusion criterion number 5 and were not separated in the analyses, as they represented only 2% of the total sample. ^d^ We contacted the authors and were unable to obtain more information regarding the other types of treatment received by the participants. ^e^ We chose to include this study, even though some participants did not meet inclusion criterion number 5 and were included in the analyses, as they represent only 10% of the total sample. ^f^ We chose to include this study, even though some participants did not meet inclusion criterion number 5 and were included in the analyses, as they represented only 2% of the total sample. ^g^ We contacted the authors and were unable to obtain more information regarding the ‘treatment types’ received by participants. ^h^ We contacted the authors and were unable to obtain more information regarding the ‘mean time since end of treatment’. *Abbreviations.* CES-D, Center for Epidemiologic Studies-Depression; EORTC QLQ-C30, European Organisation for Research and Treatment of Cancer; EORTC QLQ-PR25, European Organisation for Research and Treatment of Cancer-Prostate; FACT-B, Functional Assessment of Cancer Therapy-Breast; FACT-C, Functional Assessment of Cancer Therapy-Colorectal; FACT-G, Functional Assessment of Cancer Therapy-General; FACT-Lung, Functional Assessment of Cancer Therapy-Lung; HADS, Hospital Anxiety and Depression Scale; IPAQ, International Physical Activity Questionnaire; MAX-PC, Memorial Anxiety Scale for Prostate Cancer; PHQ-9, Patient Health Questionnaire-9; POMS, Profile of Mood States; PROMIS, Patient Reported Outcomes Measurement Information System; PTGI, Post Traumatic Growth Inventory; SAI, Spielberger’s State Anxiety Inventory; SF-36, 36-Item Short Form Health Survey; SQUASH, Short QUestionnaire to ASsess Health enhancing physical activity; SWLS, Satisfaction With Life Scale.

### 3.4. Cross-Sectional Studies (n = 16) 

With respect to the independent variables, most included cross-sectional studies assessed PA and ST using an objective measure (*n* = 14), the most popular being the ActiGraph accelerometer (*n* = 10). Four other cross-sectional studies measured PA and ST using another type of accelerometer, which was either the SenseWear Pro3 or SenseWear Mini armbands, the activPAL, or the triaxial MOX activity monitor. The two remaining studies examined PA and ST using the validated and self-reported International Physical Activity Questionnaire (IPAQ [60]) or homemade questions formulated specifically for the study.

Regarding the dependent variables, the two most frequently evaluated psychological constructs were anxiety (*n* = 6) and depression (*n* = 7). Global mental health (*n* = 1), satisfaction with life (*n* = 2), and psychological growth (*n* = 1) were the other psychological constructs assessed. It is worth noting that some studies considered multiple psychological constructs simultaneously (*n* = 6). Moreover, eight studies focused solely on QoL/health-related quality of life (HRQoL) and did not assess any of the psychological functioning constructs mentioned above. Given that QoL and HRQoL appear to be used interchangeably (e.g., Vallance et al. [55] as well as Hidde et al. [47] both used the Functional Assessment of Cancer Therapy-General questionnaire, but the former referred to it as an HRQoL questionnaire and the latter as a QoL assessment), and because QoL usually includes HRQoL [61], the term QoL is used from now on for the sake of conciseness.

#### 3.4.1. Associations of PA and ST with Anxiety and Depression Symptoms

Six studies [32,44,50,54,56,57] investigated the associations of PA and ST with anxiety and depression symptoms, and one [52] focused solely on depression. The results revealed mixed evidence. For instance, although Gaskin et al. [32] reported that MVPA had stronger associations with anxiety and depressive symptoms than ST in patients with prostate cancer, no statistically significant results were found. Conversely, Vallance et al. [54] found that meeting PA guidelines was associated with reduced anxiety symptoms, but not depression, while ST did not show significant associations with both psychological symptoms in individuals with colon cancer. Thus, despite the overall trend in the studies indicating that higher PA and lower ST levels were associated with decreased symptoms of anxiety and depression, statistical significance was not consistently observed.

#### 3.4.2. Associations of PA and ST with Psychological Constructs other Than Anxiety and Depression

Two studies conducted by Vallance et al. assessed the associations of PA and ST with satisfaction with life among 180 patients with colon cancer [54] and 127 patients with lung cancer [56], respectively. The authors found that 1) meeting PA guidelines was associated with higher satisfaction with life, while ST was unrelated to this same outcome among patients with colon cancer [54] and 2) more ST, but not MVPA, was associated with lower and moderate levels of satisfaction with life among patients with lung cancer [56]. However, in the same study [56] MVPA and ST were not significantly associated with psychological growth among these patients with lung cancer. Lastly, one study showed that both higher MVPA levels and lower ST levels were associated with greater global mental health in patients with cancer [25].

#### 3.4.3. Associations of PA and ST with QoL

Out of the 11 cross-sectional studies that investigated the associations of PA and ST with QoL [12,32,48,50,51,53,55,57,59], four found associations between higher levels of PA and global QoL [12,53,55,57]. For example, D’Silva et al. [12] showed that higher light-intensity PA levels were associated with greater QoL scores in patients with lung cancer; however, MVPA was unrelated. Although the remaining seven studies did not find associations between PA and global QoL, which is an overall score, most of them [32,46,48,50,51,59] still reported that higher levels of PA were associated with greater individual scores on various QoL subscales, including physical and functional well-being, insomnia, and bodily pain.

With regard to studies investigating the relationships between ST and QoL (*n* = 10), one [12] reported that higher ST levels were related to lower QoL, when considering a global score, but only among participants reporting the highest levels of QoL. Interestingly, three other studies examined linkages with QoL when replacing ST with PA [47,50,57]. Results showed that substituting 30 min [47,57] or one hour [50] per day of ST with equal PA time was associated with an increase in overall QoL scores. Additional studies [12,48,51] also revealed that more ST was associated with lower scores on many individual QoL subscales, such as physical, role, and cognitive functioning, as well as general health and vitality.

In the only study investigating the associations of PA and ST with QoL using a self-reported activity measure (i.e., the IPAQ [60]), Yan et al. [59] found that higher, versus lower, levels of PA were related to better physical, role, and emotional function, as well as reduced appetite loss and nausea/vomiting, while higher ST was related to lower physical function and higher insomnia, fatigue, and pain scores.

### 3.5. Longitudinal Studies (n = 4)

Out of the four included longitudinal studies [17,45,49,58], two assessed PA and ST using the ActiGraph accelerometer [17,49], while the other two examined these variables using either an adapted version of the self-reported Leisure Time Exercise Questionnaire [58] or a combination of the validated self-reported Short QUestionnaire to ASsess Health-enhancing physical activity (known as SQUASH) and the triaxial MOX accelerometer [45]. These longitudinal studies mainly investigated psychological constructs, namely anxiety (*n* = 1), depression (*n* = 2), and emotional well-being (*n* = 1), whereas only one examined QoL.

#### 3.5.1. Associations of PA and ST with Anxiety and Depression Symptoms

One study investigated the associations of PA and ST with anxiety and depression symptoms, and one focused solely on depression. While both studies targeted patients with breast cancer, they obtained somewhat inconsistent results. More specifically, Doré et al. [17] found that higher levels of MVPA were related to lower scores of depressive symptoms over a 48-month follow-up period, whereas higher levels of ST were associated with higher scores of depressive symptoms over that same period. Conversely, Phillips et al. [49] found no relationship between PA or ST and anxiety or depression symptoms over a six-month follow-up period.

#### 3.5.2. Associations of PA and ST with Psychological Constructs other Than Anxiety and Depression

Only one longitudinal study examined the relationships between PA and ST and other psychological constructs. Specifically, Wrosch et al. [58] focused on emotional well-being in breast cancer survivors and found that higher PA levels at baseline were associated with more positive emotions and less negative emotions three months later, while baseline ST was only linked to less positive emotions three months later.

#### 3.5.3. Associations of PA and ST with QoL

In the only longitudinal study investigating the associations of baseline PA and ST with future QoL, Floor Kenkhuis et al. [45] found that higher MVPA levels were independently related to greater QoL among patients with colorectal cancer over a 24-month follow-up, that is, after controlling statistically for ST, whereas higher ST levels were associated with lower QoL, beyond statistical adjustment for MVPA levels.

## 4. Discussion

This scoping review aimed to synthesize evidence regarding the associations of PA and ST with post-treatment psychological functioning and QoL in adults with cancer. The 20 included studies were mostly cross-sectional (*n* = 16), conducted from 2013–2022 in seven countries, and predominantly based on samples including a single cancer site (*n* = 17), such as breast cancer. PA and ST, conceptualized here as independent variables, were assessed mainly with accelerometers (*n* = 17), whereas psychological constructs and QoL, viewed here as dependent variables, were constantly measured with self-reported validated questionnaires (*n* = 20). Overall, while statistical significance was not consistently observed, many of the included studies indicated that higher PA levels were associated with lower anxiety and depression symptoms, as well as greater QoL levels. The inverse, whereby greater ST levels were related to greater anxiety and depression symptoms, and lower QoL levels, was often, although not always, observed.

When considering anxiety and depression symptoms, the results were not always consistent across studies. These differences may be explained by the various lengths of follow-up periods in longitudinal studies, ranging from 3–48 months, or the psychological measures used. For instance, some results were based on the Center for Epidemiologic Studies Depression Scale (also known as the CES-D), whereas others were obtained with the Hospital Anxiety and Depression Scale (also known as the HADS). While the former scale assesses a broad range of depressive symptoms, including somatic and affective ones, interpersonal difficulties, and positive affect, the latter excludes somatic items, such as dizziness and sleep disturbance, to differentiate emotional symptoms from those that could be attributed to a medical condition [62]. These scale differences could, at least partly, explain inconsistencies among the results.

Aside from anxiety and depression symptoms, a few other psychological constructs were examined in the included studies, although their number was very limited, and all but one were cross-sectional. Despite a few inconsistent results, existing studies suggest that while PA and ST were unrelated to psychological growth [56], higher PA and lower ST levels were associated with greater levels of global mental health [25], emotional well-being [58], and satisfaction with life [54,56]. However, while the existing studies, primarily focusing on psychological “distress” markers (e.g., anxiety and depression symptoms), provide valuable insights into their relationships with PA and ST, further exploration of psychological “well-being” markers (e.g., mastery, purpose in life, positive affect, life satisfaction, optimism [63,64,65]) is essential to develop a holistic perspective on psychological functioning among patients with cancer and to inform interventions that aim to promote positive psychological outcomes. In fact, psychological well-being is now recognized as not simply the absence of psychological distress [64], which highlights the importance for future work to approach psychological functioning from a more positive angle.

Results pertaining to the associations of PA and ST with QoL indicated that PA would have a more consistent association with this psychosocial indicator than ST. One possible explanation might pertain to the multidimensional nature of QoL. In fact, although ST was not always significantly associated with QoL in studies considering it an overall score, it was related to various QoL subscales separately (e.g., physical, role, and cognitive functioning), suggesting that ST–QoL linkages might be domain-specific.

Similarly, the psychological symptoms and other constructs under consideration in this scoping review appeared somewhat more consistently associated with PA than with ST. This could be explained by the fact that the included studies examined PA and ST independently rather than considering their synergistic effects. For instance, Trinh et al. [52] found that ST was associated with depression among individuals with lower PA levels, whereas this association was not significant among individuals with higher PA levels. Interestingly, these same authors found a significant, although modest, correlation between PA and ST (*r* = −0.43; *p* < 0.01), suggesting that while these two constructs are moderately correlated, they are still distinct factors, which can both influence the outcomes under study. Given that regular PA is known to release endorphins, which can reduce symptoms of anxiety and depression [66,67], it is plausible that individuals with higher PA levels may exhibit greater resistance to the adverse psychological effects of ST relative to those with lower PA levels. This hypothesis is consistent with broader health research, which has shown that high PA levels attenuate the increased risk of death associated with considerable ST [68]. In sum, since PA and ST can alter their respective associations with psychosocial outcomes, it is important for future research not only to consider these two constructs within a given study but also to consider their synergistic effect.

Finally, it is worth mentioning that among the eight studies included in this scoping review that examined clinical significance [25,32,45,47,49,53,55,57], six of them [25,32,45,47,49,53] reported clinical improvements (for details see Supplementary Table S1). In a nutshell, it is essential to acknowledge that achieving clinically significant changes may require substantial increases in PA or decreases in ST, emphasizing the need for nuanced interpretations. For example, Welch et al. [57] found that in order to reach clinical significance for QoL, one would have to substitute about 70 min of ST for MVPA per day, while Gaskin et al. [32] revealed that an increase in MVPA of about one hour per day was needed to reach clinical significance in QoL subscales. However, more studies examining clinical improvements, in addition to statistical significance, are needed for a more comprehensive and meaningful interpretation of the research findings.

### 4.1. Gaps in the Literature

The literature presented in this scoping review has various strengths, including the use of objective PA/ST measures in most studies (*n* = 17 out of 20; 85%). Accelerometers provide objective, precise, and reliable measurements of movement patterns throughout the day, which is not always the case for subjective measures. Indeed, while self-reporting methods are frequently used due to their practicality, low cost, low participant burden, and general acceptance [69], they can also over- or underestimate true PA energy expenditure and rates of inactivity [70]. Moreover, self-reporting measures are also subject to recall problems and response bias (e.g., social desirability and inaccurate memory) and the inability to capture the absolute levels of PA and ST [70]. A second strength of this scoping review is the relatively large sample sizes (median = 177) reported in the included studies, which reinforces the generalizability of the research results. Only one study [51] had a small sample size, with 20 participants.

However, the studies included in this scoping review present some limitations that are worth mentioning. First, most of the included studies were cross-sectional and correlational (*n* = 16), and none were interventional, experimental, or quasi-experimental. The disadvantages of these study designs include their inability to document changes in constructs of interest over time, and to confirm causal inferences. Second, the included studies relied on heterogenous measures (e.g., distinct depression measures), construct definitions, selected cancer period (e.g., closer vs. further apart from diagnosis), and samples (e.g., single vs. mixed cancer sites), among others, which makes it harder to draw clear conclusions. For instance, knowing that systematic reviews have shown discrepancies when comparing self-reported with objectively assessed PA [70] and ST [71], future studies should use both objective and subjective measures to assess PA and ST in order to compare the associations of these two variables with the psychological functioning and QoL of patients with cancer. With regard to the selected cancer period, it is most likely that individuals’ PA and ST levels vary considerably closer versus further apart from diagnosis and treatment termination, which, in turn, may influence their psychological functioning and QoL. Third, as discussed above, subjective measures were rarely used in studies included in this review (*n* = 4 out of 20; 20%), which precludes the comparison of associations obtained between objective vs. subjective PA/ST measures and psychosocial outcomes. Despite the potential issues related to the use of subjective measures noted in the prior paragraph, these measures still provide valuable, complementary information to the ones obtained via objective measures, as they capture individuals’ perception of their PA/ST levels, which can be intervention targets themselves. Fourth, most of the included studies examined PA and ST separately, although it has been demonstrated that they have a synergistic influence on psychological functioning and QoL [47,64]. Altogether, these limitations highlight the relevance of using, for instance, experimental and quasi-experimental longitudinal designs among homogeneous samples to evaluate psychological functioning and QoL at specific time points in the post-treatment period. This methodology would certainly allow researchers to better understand the unique and interactive associations of PA and ST with subsequent psychological functioning and QoL among patients with cancer.

### 4.2. Future Avenues

Although the studies included in this scoping review provided important insights into the associations of PA and ST with cancer survivors’ psychological functioning and QoL over the post-treatment period, none of them examined these outcomes using daily life measurements, such as ecological momentary assessment (EMA) or daily diaries. The former consists of survey notifications sent to participants every few minutes to hours, with items answered at the moment, while the latter entails surveys completed once per day, with items answered retrospectively. Some of the advantages of EMA and daily diaries over traditional questionnaires include stronger ecological validity, lower recall bias, and the ability to address research questions on within-person processes—that is, processes that unfold within individuals over time [72,73,74].

These research designs appear beneficial for studying both PA/ST and psychosocial variables. In fact, many movement patterns, including PA/ST, and psychological constructs, such as anxiety and depression symptoms, can fluctuate throughout the day and from one day to another in response to specific contexts. Undoubtedly, EMA and daily diaries would allow researchers to capture these dynamic processes by assessing variables repeatedly over time. This could provide a better understanding of how movement patterns and psychological constructs change among individuals as a result of their environmental contexts.

It is worth noting that EMA and daily diaries have been found to be feasible and useful in oncology research and are deemed to hold unique promise to gain a better understanding of patients’ QoL and disease- and treatment-related problems [75,76]. Accordingly, a recent scoping review [76] identified 12 studies using EMA among patients with cancer, but none combined objective PA monitoring with EMA to measure movement patterns and psychosocial indicators. To our knowledge, no study has examined the associations between objectively assessed PA and ST and the psychological functioning and/or QoL of adults with cancer in the post-treatment period using EMA or daily diaries. However, there is a critical need to understand the real-time relationship between PA, ST, and psychological functioning and QoL to refine recommendations and motivate patients to be physically active.

Finally, future work should also provide information about the context of PA (e.g., alone/in group and indoor/outdoor) and ST (e.g., computer use and driving) since these distinctions may influence the effects of PA/ST on psychosocial constructs. For instance, it has been suggested that some sedentary behaviours (e.g., watching television) can distance individuals from social interactions and increase their risk of depression [77]. However, other sedentary behaviours may promote social interactions (e.g., conversing with a friend) or well-being (e.g., reading a book) and are thus nonetheless beneficial for psychological functioning [78]. With regard to the context in which PA is performed, a literature review among female cancer survivors showed that although both indoor and outdoor PA settings elicit positive effects, compared to no exercise, outdoor environments stimulate more positive affective responses, such as greater decreases in anxiety and depression symptoms [79].

### 4.3. Strengths and Limitations of the Current Scoping Review

The main strength of this scoping review is its use of a rigorous and comprehensive search strategy to address its objectives. However, one of its limitations is that, while the search was designed to be exhaustive, some articles published in other databases may not have been included or identified by our search terms. Moreover, including only English and peer-reviewed published articles may have limited the inclusion of a few potentially relevant non-English and grey literature studies. Finally, the quality appraisal and risk of bias in published studies were not conducted, as they are deemed unnecessary for scoping reviews [41].

### 4.4. Clinical Implications

The benefits of PA are now recognized by most oncologists and patients; yet the integration of PA into the medical care of patients is still not widely endorsed [80]. Indeed, a recent study revealed that while 88% of patients with cancer had heard about the benefits of PA, only 11% had received a prescription from their oncologist [80]. It is possible that the divergent results found across studies regarding the associations between PA, ST, and psychosocial functioning in post-treatment patients with cancer is difficult for healthcare professionals to interpret, and further discourage them from recommending an increase in PA as well as a decrease in ST to their patients. By summarizing the results of the current literature, this scoping review underlines that while there is some suggestion that higher PA and lower ST levels are associated with better psychosocial outcomes, additional studies are warranted because the evidence remains somewhat mixed. As this body of research continues to grow, it will serve as a scientific foundation when it comes to a better comprehension of the potential benefits regarding increased PA and reduced ST in adults with cancer. Moreover, longitudinal studies revealing temporal associations between PA, ST, and psychosocial functioning underline the importance of ongoing assessment and follow-up. Therefore, healthcare providers should consider regular evaluations to monitor changes in movement patterns and psychosocial functioning over time among post-treatment patients with cancer.

## 5. Conclusions

The present scoping review reports on the associations of PA and ST with psychological functioning and QoL among adults with cancer in the post-treatment period. While informative, the identified studies were mainly cross-sectional, and their results showed some discrepancies, which could possibly be explained by differences in the methodologies used. Consequently, future research should favor longitudinal designs and use EMA or daily diaries to assess proximal, dynamic associations of PA/ST with future psychological functioning and QoL outcomes. Subsequent work must also consider PA’s and ST’s interaction effects in predicting such psychosocial indicators. That being said, many results from the current review combined with prior available scientific evidence suggest that healthcare providers should educate patients about the interrelations between PA, ST, psychological functioning, and QoL, since promoting PA and reducing ST have the potential to yield psychosocial benefits.

## Figures and Tables

**Table 1:** Search strategy for this scoping review.

Concept	Search Number	Terms
Post-treatment	1	(“After treatment*” OR “After-Treatment*” OR Recovery OR “Post-treatment*” OR Posttreatment* OR “Post Treatment” OR Surviv* OR “Follow-Up” OR Aftercare OR “After Care”) N4 ^a^ Cancer*
Physical activity (PA)	2	Sport* OR “Physical Activit*” OR “Physical-activit*” OR Exercise* OR “Physical Exercise*” OR Training OR “Physical training” OR Fitness OR Workout* OR Aerobic
Sedentary time (ST)	3	Sedentar*
Psychological functioning/QoL	4	“Quality Of Life” OR “Life Quality” OR QoL OR HRQoL OR “Well Being” OR “Well-being” OR Affect* OR Emotion* OR Mood* OR Feeling* OR “Mental-health” OR “Mental Health” OR Anxiet* OR Depress* OR Stress OR Distress*
Combined search	5	1 AND 2 AND 3 AND 4

^a^ “N4” is used as a proximity operator. It specifies the allowable word distance between terms or phrases on either side.

## Data Availability

The original contributions presented in the study are included in the article/Supplementary Material, further inquiries can be directed to the corresponding author.

## Supplementary Materials

The following supporting information can be downloaded at: https://www.mdpi.com/article/10.3390/ijerph21020225/s1, Table S1: Summary of key findings for each included study (*n* = 20).
