# Supplementary material for: Physical Activity, Sedentary Time, and Psychosocial Functioning among Adults with Cancer: A Scoping Review
Source: Int J Environ Res Public Health. 2024 Feb 14;21(2):225. doi: 10.3390/ijerph21020225 (PMC10888167; doi:10.3390/ijerph21020225)
Supplement: Supplementary file 1 [file ijerph-21-00225-s001.zip › ijerph-2810765-supplementary.pdf]

Supplementary Table S1

Summary of key findings for each included study (n = 20)

First Author, Year of Publication	Study Design	Key Findings
Di Blasio et al., 2022 [1]	Cross-sectional	Higher ST levels were associated with higher anxiety and depression symptoms, while PA exhibited opposite associations.
Doré et al., 2022 [2]	Longitudinal over 48 months<br><br>(Every 3 months during the first year, and then after 24 and 48 months)	MVPA and ST were independent predictors of depressive symptoms. Higher levels of MVPA were associated with lower scores of depressive symptoms, whereas higher levels of ST were associated with higher scores of depressive symptoms. The interaction between MVPA and ST was non-significant.
D'Silva et al., 2018 [3]	Cross-sectional	Total MVPA was not associated with QoL. Higher levels of total light-intensity PA were associated with higher QoL scores at the 50th and 75th percentiles and higher physical and functional well-being scores at the 50th and 75th percentiles. Higher levels of total ST were associated with lower QoL scores at the 75th percentile and lower physical and functional well-being scores at the 25th, 50th, and 75th percentiles.
Floor Kenkhuis et al., 2021 [4]	Longitudinal over 24 months<br><br>(6 weeks, 6 months, 12 months, and 24 months post-treatment)	Higher levels of ST were associated with lower QoL scores. Similarly, higher levels of MVPA were independently associated with higher QoL scores over time, even when accounting for ST. Associations were also clinically significant.
Gaskin et al., 2016 [5]	Cross-sectional	MVPA had stronger associations with QoL, anxiety, and depressive symptoms than ST, but no statistically significant results were found. However, increasing MVPA by less than 1 h/day showed clinically important differences in symptom scales, and meeting recommended MVPA levels had potential clinical benefits for insomnia and financial difficulties.
Hartman et al., 2017 [6]	Cross-sectional	Higher levels of ST were associated with lower physical health scores, but this association became non-significant when adjusting for MVPA, whereas MVPA itself was a significant independent predictor of physical health scores. ST was not associated with mental health, and there was no evidence of moderation by MVPA for the association between ST and QoL.
Hidde et al., 2022 [7]	Cross-sectional	Reallocating 30 minutes of ST to MVPA was associated with higher QoL scores. No significant changes were observed for the QoL subscales. A clinically meaningful increase in QoL scores was observed for reallocating 30 minutes of ST to MVPA or 30 minutes of light PA to MVPA. A clinically meaningful decrease in QoL scores was observed when reallocating time from MVPA to light PA or ST.

First Author, Year of Publication	Study Design	Key Findings
Nurnazahiah et al., 2022 [8]	Cross-sectional	MVPA was associated with global health status, whereas higher ST levels were associated with lower physical functioning, role functioning, and cognitive functioning scores.
Phillips et al., 2015 [9]	Longitudinal over 6 months<br><br>(Baseline and 6 months later)	Higher levels of baseline MVPA were associated with higher physical well-being, and total FACT-B scores, as well as fewer breast cancer-specific concerns at six months, and these relationships remained largely unchanged after controlling for ST. Baseline ST was associated with lower physical well-being scores at six months, but the relationship did not hold when MVPA was taken into account. Compared with those with lower MVPA levels at baseline, survivors with higher MVPA levels had clinically meaningful higher QoL at six months.
Rees-Punia et al., 2020 [10]	Cross-sectional	Higher levels of MVPA and lower levels of ST were associated with higher mean scores for global mental health. The differences in the mean scores were clinically meaningful.
Roekel et al., 2016 [11]	Cross-sectional	PA was associated with higher physical functioning and lower disability scores. A significantly higher physical functioning score was observed for substituting 1 h/day of ST with PA. Favorable associations surpassing medium effect size cut-offs were observed for substituting 1 h/day of ST with equal time in PA for global QoL, disability, depression, and anxiety scores, but these associations did not reach statistical significance.
Schofield et al., 2018 [12]	Cross-sectional	MVPA was associated with higher physical functioning, general health, and physical component summary scores, as well as lower bodily pain. Higher ST levels were associated with lower physical functioning, general health, vitality, and mental health scores.
Trinh et al., 2015 [13]	Cross-sectional	ST predicted higher levels of depression, and light-intensity PA predicted lower levels of depression. There was a significant interaction effect between MVPA and ST on depression, where ST had a stronger negative impact on this outcome for individuals with lower levels of MVPA, while it did not significantly affect those with higher levels of MVPA.
Vallance et al., 2014 [14]	Cross-sectional	Significant differences in FACT-C scores were found across quartiles of MVPA. Participants meeting MVPA guidelines reported better QoL scores compared to those who did not meet the guidelines. No significant trends were observed for ST.
Vallance et al., 2015 [15]	Cross-sectional	Meeting PA guidelines was associated with reduced anxiety symptoms and higher satisfaction with life. ST did not show significant differences in psychological health outcomes.
Vallance et al., 2017 [16]	Cross-sectional	MVPA in bouts of 10+ minutes was associated with higher QoL scores. No significant associations were found between ST and QoL. No clinically significant associations for ST, light PA or MVPA emerged for QoL.

First Author, Year of Publication	Study Design	Key Findings
Vallance et al., 2018 [17]	Cross-sectional	Higher levels of ST and lower levels of total light-intensity PA were associated with higher depressive symptoms at the 50th percentile and lower satisfaction with life at the 25th and 50th percentiles. However, total MVPA was not significantly associated with any of the assessed psychosocial health outcomes.
Welch et al., 2019 [18]	Cross-sectional	MVPA was associated with higher scores in the FACT-B total, physical well-being, and functional well-being scores. Reallocating 30 minutes of ST to MVPA was associated with higher scores in the FACT-B total, physical well-being, and functional well-being scores; however, no significant associations were found between PA and anxiety or depression. None of the reallocation change scores reached clinical significance.
Wrosch et al., 2013 [19]	Longitudinal over 3 months<br><br>(Baseline and 3 months later)	Higher T1 levels of PA were associated with more positive affect, less negative affect, and fewer physical symptoms (at both T1 and T2). Higher ST levels (at both T1 and T2) were associated with less positive affect at T2.
Yan et al., 2021 [20]	Cross-sectional	Higher levels of PA were associated with higher physical, role, and emotional function scores, as well as reduced appetite loss and nausea/vomiting compared to lower levels of PA, while higher ST levels were associated with lower physical function and higher insomnia, fatigue, and pain scores.

Abbreviations. FACT-B, Functional Assessment of Cancer Therapy-Breast; FACT-C, Functional Assessment of Cancer Therapy-Colorectal; MVPA, Moderate-to-vigorous physical activity; PA, Physical activity; QoL, Quality of life; ST, Sedentary time.

References

1. Di Blasio, A.; Di Dalmazi, G.; Morano, T.; Bucci, I.; Verrocchio, S.; Grossi, S.; Cianchetti, E.; Valentini, P.; Cugusi, L.; Gobbo, S.; et al. Relationships between daily physical activity combinations and psychophysical health status of Italian breast cancer survivors. *Home Health Care Serv. Q.* **2022**, *41*, 200–218. https://doi.org/10.1080/01621424.2022.2041524.
2. Doré, I.; Plante, A.; Peck, S.S.; Bedrossian, N.; Sabiston, C.M. Physical activity and sedentary time: Associations with fatigue, pain, and depressive symptoms over 4 years post-treatment among breast cancer survivors. *Support. Care Cancer* **2022**, *30*, 785–792. https://doi.org/10.1007/s00520-021-06469-2.
3. D'Silva, A.; Gardiner, P.A.; Boyle, T.; Bebb, D.G.; Johnson, S.T.; Vallance, J.K. Associations of objectively assessed physical activity and sedentary time with health-related quality of life among lung cancer survivors: A quantile regression approach. *Lung Cancer* **2018**, *119*, 78–84. https://doi.org/10.1016/j.lungcan.2018.03.010.
4. Floor Kenkhuis, M.; Van Roekel, E.H.; Breedveld-Peters, J.J.L.; Breukink, S.O.; Janssen-Heijnen, M.L.G.; Keulen, E.T.P.; Van Duijnhoven, F.J.B.; Mols, F.; Weijenberg, M.P.; Bours, M.J.L. Longitudinal Associations of Sedentary Behavior and Physical Activity with Quality of Life in Colorectal Cancer Survivors. *Med. Sci. Sports Exerc.* **2021**, *53*, 2298–2308. https://doi.org/10.1249/MSS.0000000000002703.
5. Gaskin, C.J.; Craike, M.; Mohebbi, M.; Salmon, J.; Courneya, K.S.; Broadbent, S.; Livingston, P.M. Associations of objectively measured moderate-to-vigorous physical activity and sedentary behavior with quality of life and psychological well-being in prostate cancer survivors. *Cancer Causes Control* **2016**, *27*, 1093–1103. https://doi.org/10.1007/s10552-016-0787-5.
6. Hartman, S.J.; Marinac, C.R.; Bellettiere, J.; Godbole, S.; Natarajan, L.; Patterson, R.E.; Kerr, J. Objectively measured sedentary behavior and quality of life among survivors of early stage breast cancer. *Support. Care Cancer* **2017**, *25*, 2495–2503. https://doi.org/10.1007/s00520-017-3657-0.
7. Hidde, M.C.; Lyden, K.; Henry, K.; Leach, H.J. Reallocating time to physical activity and sleep: Associations with quality of life in cancer survivors. *Support. Care Cancer* **2022**, *30*, 7527–7534. https://doi.org/10.1007/s00520-022-07187-z.
8. Nurnazahiah, A.; Shahril, M.R.; Nor Syamimi, Z.; Ahmad, A.; Sulaiman, S.; Lua, P.L. Relationship of objectively measured physical activity and sedentary behaviour with health-related quality of life among breast cancer survivors. *Health Qual. Life Outcomes* **2020**, *18*, 222. https://doi.org/10.1186/s12955-020-01478-x.
9. Phillips, S.M.; Awick, E.A.; Conroy, D.E.; Pellegrini, C.A.; Mailey, E.L.; McAuley, E. Objectively measured physical activity and sedentary behavior and quality of life indicators in survivors of breast cancer. *Cancer* **2015**, *121*, 4044–4052. https://doi.org/10.1002/cncr.29620.

10. Rees-Punia, E.; Patel, A.V.; Nocera, J.R.; Chantaprasopsuk, S.; Demark-Wahnefried, W.; Leach, C.R.; Smith, T.G.; Cella, D.; Gapstur, S.M. Self-reported physical activity, sitting time, and mental and physical health among older cancer survivors compared with adults without a history of cancer. *Cancer* **2020**, *127*, 115–123. https://doi.org/10.1002/cncr.33257.
11. Roekel, E.; Bours, M.; Breedveld-Peters, J.; Willems, P.; Meijer, K.; Kant, I.; den Brandt, P.; Beets, G.; Sanduleanu, S.; Weijenberg, M.; et al. Modeling how substitution of sedentary behavior with standing or physical activity is associated with health-related quality of life in colorectal cancer survivors. *Cancer Causes Control* **2016**, *27*, 513–525. https://doi.org/10.1007/s10552-016-0725-6.
12. Schofield, C.; Newton, R.U.; Cohen, P.A.; Galvão, D.A.; McVeigh, J.A.; Mohan, G.R.; Tan, J.; Salfinger, S.G.; Straker, L.M.; Peddle-McIntyre, C.J. Health-related quality of life and pelvic floor dysfunction in advanced-stage ovarian cancer survivors: Associations with objective activity behaviors and physiological characteristics. *Support. Care Cancer* **2018**, *26*, 2239–2246. https://doi.org/10.1007/s00520-018-4069-5.
13. Trinh, L.; Amireault, S.; Lacombe, J.; Sabiston, C.M. Physical and psychological health among breast cancer survivors: Interactions with sedentary behavior and physical activity. *Psychooncology* **2015**, *24*, 1279–1285. https://doi.org/10.1002/pon.3872.
14. Vallance, J.K.; Boyle, T.; Courneya, K.S.; Lynch, B.M. Associations of objectively assessed physical activity and sedentary time with health-related quality of life among colon cancer survivors. *Cancer* **2014**, *120*, 2919–2926. https://doi.org/10.1002/cncr.28779.
15. Vallance, J.K.; Boyle, T.; Courneya, K.S.; Lynch, B.M. Accelerometer-assessed physical activity and sedentary time among colon cancer survivors: Associations with psychological health outcomes. *J. Cancer Surviv.* **2015**, *9*, 404–411. https://doi.org/10.1007/s11764-014-0409-8.
16. Vallance, J.K.; Buman, M.P.; Lynch, B.M.; Boyle, T. Reallocating time to sleep, sedentary, and active behaviours in non-Hodgkin lymphoma survivors: Associations with patient-reported outcomes. *Ann. Hematol.* **2017**, *96*, 749–755. https://doi.org/10.1007/s00277-017-2942-9.
17. Vallance, J.K.; Bebb, G.D.; Boyle, T.; Johnson, S.T.; Gardiner, P.A.; D'Silva, A. Psychosocial health is associated with objectively assessed sedentary time and light intensity physical activity among lung cancer survivors. *Ment. Health Phys. Act.* **2018**, *14*, 61–65. https://doi.org/10.1016/j.mhpa.2018.02.002.
18. Welch, W.A.; Ehlers, D.; Gavin, K.L.; Aguinaga, S.; Cottrell, A.; Nielsen, A.; Solk, P.; McAuley, E.; Phillips, S.M. Effects of reallocating sedentary time with physical activity on quality of life indicators in breast cancer survivors. *Psychooncology* **2019**, *28*, 1430–1437. https://doi.org/10.1002/pon.5091.
19. Wrosch, C.; Sabiston, C.M. Goal adjustment, physical and sedentary activity, and well-being and health among breast cancer survivors. *Psychooncology* **2013**, *22*, 581–589. https://doi.org/10.1002/pon.3037.
20. Yan, R.; Che, B.; Lv, B.; Wu, P.; Lu, X.; Zhang, Y.; Wang, J.; Yu, J. The association between physical activity, sedentary time and health-related quality of life in cancer survivors. *Health Qual. Life Outcomes* **2021**, *19*, 213. https://doi.org/10.1186/s12955-020-01575-x.
